# INFLAMM-AGING IS NOT A UNIVERSAL AGING MECHANISM ACROSS DIVERSE HUMAN POPULATIONS

**DOI:** 10.1093/geroni/igae098.2292

**Published:** 2024-12-31

**Authors:** Maximilien Franck, Kamaryn Tanner, Luigi Ferrucci, Michael Gurven, Ameneh Mehrjerd, Joe Yeong, Johannes Hertel, Alan A Cohen

**Affiliations:** University of Sherbrooke, Sherbrooke, Quebec, Canada; Columbia University, New York, New York, United States; National Institute on Aging, Baltimore, Maryland, United States; University of California Santa Barbara, Santa Barbara, California, United States; University Medicine Greifswald, Greifswald, Mecklenburg-Vorpommern, Germany; Institute of Molecular and Cell Biology, Singapore, Singapore; University Medicine Greifswald, Greifswald, Mecklenburg-Vorpommern, Germany; Columbia University, New York, New York, United States

## Abstract

Inflamm-aging, defined by age-associated increases in inflammatory cytokines, is thought to be a universal mammalian aging mechanism associated with higher chronic disease risk, as observed in industrialized populations like the InCHIANTI cohort (Italy). However, consensus regarding its precise nature and a measurement framework is lacking. Its relevance and measurement applicability across diverse human populations, especially in non-industrialized societies with high infection burdens, remain unclear. We aimed to characterize inflamm-aging across four populations, examining its relation to age and chronic diseases. Through principal components and factor analysis, we studied immune variation within the InCHIANTI, Singapore Longitudinal Ageing Study (SLAS), Study of Health in Pomerania (SHIP), and Tsimane Health and Life History Project (THLHP) cohorts—Amerindian forager-horticulturalists from the Bolivian Amazon. We compared immune axes’ relationships with age and chronic diseases across cohorts. We observed major differences in the structure of inflammatory and immune variation across populations. Notably, the primary immune axis in the THLHP cohort diverged significantly from those in the InCHIANTI, SLAS and SHIP cohorts, with the latter two aligning more closely with the inflamm-aging phenomenon—albeit with variations in their manifestations. Furthermore, these axes did not consistently correlate with age across cohorts and exhibited differential impacts on chronic diseases, with the primary axes identified in InCHIANTI and SLAS showing stronger associations with chronic diseases. Our findings suggest inflamm-aging is not universal. There appear to be significant environmental and lifestyle impacts on inflammatory and immune variation leading to differences in how inflammation drives disease susceptibility across populations.

